# Mucopolysaccharidosis type II zebrafish model exhibits early impaired proteasomal-mediated degradation of the axon guidance receptor Dcc

**DOI:** 10.1038/s41419-024-06661-2

**Published:** 2024-04-16

**Authors:** Rosa Manzoli, Lorenzo Badenetti, Matteo Bruzzone, Maria Carla Macario, Michela Rubin, Marco Dal Maschio, Antonella Roveri, Enrico Moro

**Affiliations:** 1 Department of Molecular Medicine, University of Padova, 35121 Padova, Italy; 2 Department of Biology, University of Padova, 35121 Padova, Italy; 3 Department of Women’s and Children’s Health, University of Padova, 35128 Padova, Italy; 4 Istituto di Ricerca Pediatrica “Città Della Speranza”, 35127 Padova, Italy; 5 Department of Biomedical Sciences, University of Padova, 35121 Padova, Italy; 6 Padua Neuroscience Center - PNC, University of Padova, 35129 Padova, Italy

**Keywords:** Cell biology, Mechanisms of disease

## Abstract

Most of the patients affected by neuronopathic forms of Mucopolysaccharidosis type II (MPS II), a rare lysosomal storage disorder caused by defects in iduronate-2-sulfatase (IDS) activity, exhibit early neurological defects associated with white matter lesions and progressive behavioural abnormalities. While neuronal degeneration has been largely described in experimental models and human patients, more subtle neuronal pathogenic defects remain still underexplored. In this work, we discovered that the axon guidance receptor Deleted in Colorectal Cancer (Dcc) is significantly dysregulated in the brain of *ids* mutant zebrafish since embryonic stages. In addition, thanks to the establishment of neuronal-enriched primary cell cultures, we identified defective proteasomal degradation as one of the main pathways underlying Dcc upregulation in *ids* mutant conditions. Furthermore, *ids* mutant fish-derived primary neurons displayed higher levels of polyubiquitinated proteins and P62, suggesting a wider defect in protein degradation. Finally, we show that *ids* mutant larvae display an atypical response to anxiety-inducing stimuli, hence mimicking one of the characteristic features of MPS II patients. Our study provides an additional relevant frame to MPS II pathogenesis, supporting the concept that multiple developmental defects concur with early childhood behavioural abnormalities.

## Introduction

Mucopolysaccharidosis type II, also known as Hunter syndrome (OMIM #309900), is a rare X-linked lysosomal storage disorder (LSD) caused by defective iduronate-2-sulfatase (IDS) activity. Residing in the acidic environment of the lysosome, IDS operates in the first step of the heparan and dermatan sulfates (HS/DS) degradative cascades so that, in MPS II pathological conditions, glycosaminoglycans metabolism is significantly affected. This results in the abnormal accumulation of undegraded substrates, a characteristic feature of the lysosomal storage pathologies. However, even if traditionally MPS aetiology has been primarily attributed to lysosomal engorgement, recent evidence pointed out that autophagic flux impairment and signalling pathways dysregulation arise even before evident storage, highlighting the central role of this organelle in the maintenance of cellular homoeostasis [1–3].

Since IDS is ubiquitously expressed, all cell types may suffer from lysosomal dysfunction therefore resulting in a wide spectrum of symptoms. In this context, MPS II patients are usually grouped as *non-neuronopathic* and *neuronopathic* based on the absence or occurrence of central nervous system (CNS) involvement, respectively. In the second and more severe case, patients display cognitive impairment, developmental delay and progressive neurocognitive regression in addition to somatic manifestations [4]. These symptoms are frequently paralleled by CNS anatomical defects: indeed, patients may present focal or diffuse white matter lesions, progressive demyelination and hydrocephalus [5]. CNS-related abnormalities frequently arise by 4 years of age but can be anticipated to the first year of age in rapidly progressing cases, making MPS II a disease with paediatric onset [6]. Regardless of the onset timing, this condition is extremely life-threatening: indeed MPS standard treatment, the enzyme replacement therapy (ERT), is highly ineffective in treating the CNS pathology due to the inability of the recombinant enzyme to cross the blood–brain barrier. Recently, a novel ERT has been approved in Japan: in *Pabinafusp alpha* formulation, the recombinant IDS can reach the brain parenchyma through transcytosis thanks to the humanized anti-human transferrin receptor antibody [7]. Even if this most recent approach is extremely promising, the understanding of MPS II neuropathogenesis remains still highly demanding to develop efficient pharmacological therapies.

Recently, a bulk RNA-seq analysis of MPS II mouse brain at 9 months has pointed out significant variations in the expression of proteins implicated in fundamental processes such as synapse communication, calcium signalling and axon guidance [8]. Among axon guidance cues, Deleted in Colorectal Cancer (Dcc) is one of the key players involved in the regulation of axon navigation during development. It is a transmembrane receptor usually found at the growth cone of elongating axons; when bound to its ligand Netrin, Dcc mediates axon turning towards the ligand source, thus representing a fundamental axis involved in chemoattraction. In addition, it has been demonstrated both in silico and in vivo that HS is directly recruited to mediate the interaction between Netrin and Dcc, underlying the role of HS in the modulation of signalling pathway activation [9–11]. Moreover, Dcc mutations have been associated with human neuro-pathologies such as Congenital Mirror Movement (CMM) and Developmental Split-Brain syndrome in which patients display defects in the axons of the cortico-spinal tract, anomalies in commissure fasciculation and partial agenesis of the *corpus callosum* [12]. This structure is a thick fascicle of commissural fibres involved in hemispheres communication and represents one of the most frequently affected CNS structures in MPS II patients [5].

The precise temporal and spatial regulation of axon guidance molecules represents a crucial aspect of correct signalling pathway activation, thus impacting on physiological assembly of brain circuits and their activity. For this reason, transmembrane receptors such as Dcc experience an intense shuffling between the cytosolic and membrane compartments. These movements are generally overseen by the endocytic pathway: Dcc co-localization with endosomal markers Rab11, Rab5 and Rab7 (member RAS oncogene family 11, 5 and 7) suggests that this receptor may be either recycled and relocated or targeted to the endo-lysosomal degradative pathway [13]. Furthermore, it has been demonstrated that Dcc protein levels can be additionally regulated by the ubiquitin system, and Netrin can induce Dcc degradation through the ubiquitin-proteasome axis in murine embryonic cortical neurons [14].

Given the central role of lysosomes in vesicle trafficking, signalling pathway modulation and protein degradation [15], in this study we sought to investigate the relationship between axon guidance molecules and Ids deficiency during neuronal development. We demonstrate that marked Dcc alterations are detected since embryonic stages in the brain of *ids* mutant zebrafish larvae. Moreover, using neuronal-enriched primary zebrafish cell cultures, we provide evidence that Dcc dysregulation is mainly restricted to neurons which also display defective growth behaviour. Finally, we show that Dcc dysregulation in Ids-deprived cells could be due, at least in part, to defective proteasomal degradation. Collectively, these data define axonal guidance defects as an early hallmark of neuronal dysfunction in MPS II and provide a proof-of-concept that lysosomal defects can severely impinge on key neuronal functions during embryonic stages.

## Results

### Dcc dysregulation is traceable since embryonic stages in *ids* mutant zebrafish

We previously characterized the stable *ids* mutant zebrafish line showing early molecular alterations before the onset of skeletal abnormalities [16]. However, no data are available concerning central nervous system-related defects yet. Since lysosomes play an essential role during tissue morphogenesis, especially during neuronal development [12, 17, 18], we aimed to verify whether molecular defects could be traced already at embryonic stages in the brain of MPS II fish.

**Fig. 1:**
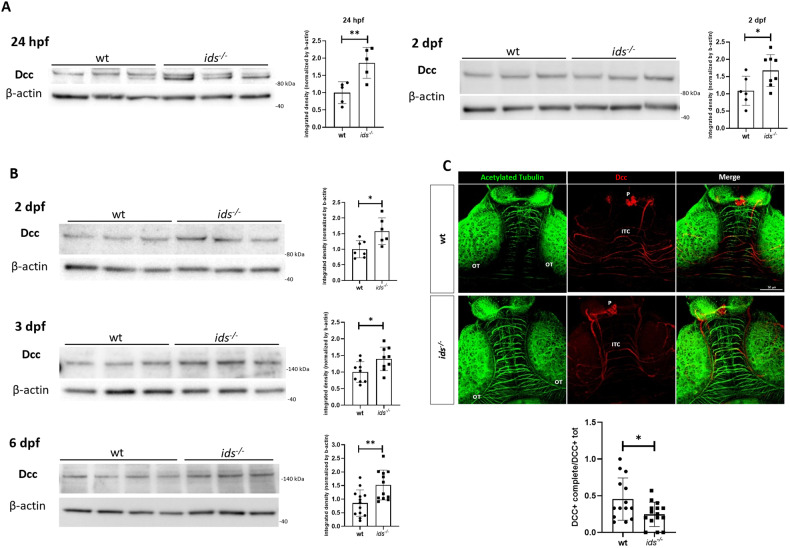
Dcc is significantly upregulated in *ids* mutant since embryonic stages. **A** Representative western blot for Dcc at 24 hpf (left) and 2 dpf (right). In both cases, lysates from whole dissected heads showed Dcc upregulation in *ids* mutant fish-derived samples with respect to wild-type ones (*n* = at least 5 biological replicates, pools of 20 dissected heads). **B** Western blot analysis of Dcc protein levels on extracted brains at 2, 3 and 6 dpf. Consistently, Dcc upregulation is significantly observed in *ids* mutant conditions when compared to controls (*n* = at least 6 biological replicates, pools of 20 dissected brains). From 3 dpf, Dcc showed a strong immunoreactive band at 140 kDa. **C** Whole-mount immunofluorescence on 6 dpf dissected brains. Acetylated tubulin was used to mark axonal processes in the midbrain region. The bar graph below shows the ratio between the number of axons displaying Dcc immunoreactivity in the entire length (Dcc+ complete) and the total Dcc-positive commissures (Dcc+ tot) for each genotype (*n* = 14 biological replicates). Dorsal view, anterior to the top. ITC intertectal commissures, OT optic tectum, P pineal gland. Scale bar: 50 μm. Data are expressed as the mean ± SD (*p < 0.05; **p < 0.005; *t*-test).

In particular, we investigated whether *ids* loss of function might impinge on Dcc homoeostasis. We firstly evaluated Dcc protein levels in mutant fish by western blot analysis: protein lysates derived from dissected heads at 24 hours post-fertilization (hpf) and 2 days post-fertilization (dpf) showed significant Dcc upregulation in *ids* mutant embryos with respect to controls (Fig. 1A). To assess whether the quantitative difference of Dcc protein levels detected in *ids* mutants could be precisely related to the brain, we moved our focus from whole heads to dissected brains. Consistently with what was previously observed, Dcc upregulation was detected in mutant fish-derived brain lysates at 2 dpf, as well as at 3 and 6 dpf (Fig. 1B), underlying that Dcc imbalance was not only restricted to the embryonic brain but it was maintained up to larval stages. In addition, to spatially localize Dcc-positive structures, we performed whole-mount immunofluorescence on control and MPS II dissected brains at 6 dpf (Fig. 1C): a positive staining was traceable around blood vessels, pineal gland and intertectal commissures, a series of big axonal tracts involved in interhemispheric communication. By looking at these midbrain fascicles by 3D reconstructions, we found that, while in control fish Dcc was localized on the entire axonal tract, in mutants these commissures resulted positive for Dcc staining only in the lateral and not in the medial region. Accordingly, the ratio between the number of axons displaying Dcc immunoreactivity along the entire length and the total Dcc-positive commissures was significantly different between mutant and control brains. In conclusion, our analysis showed that Dcc imbalance could be found in the brain of *ids* mutants since embryonic stages and that this dysregulation is maintained up to 6 dpf. Moreover, at this time point, we also retrieved a differential Dcc localization along intertectal commissures in *ids* mutant brains.

### Neuronal-enriched primary cell cultures confirm Dcc dysregulation in *ids* mutant conditions

To assess whether Dcc upregulation in *ids* mutants might be due in particular to the neuronal population, we established an optimized protocol for zebrafish-derived neuronal-enriched cell cultures starting from embryonic dissected brains. Primary neurons showed the formation of protrusions already 1 hour after seeding and, by 2 days in vitro (DIV), they exhibited a dense network of connections (Supplementary Fig. 1A). Since astroglial cells represent the most abundant glial cell type in the zebrafish brain and are known to express Dcc [19], we checked for Gfap-positive (glial fibrillary acidic protein, Gfap+) cells abundance in primary cultures. As depicted in Supplementary Fig. 1B, immunofluorescence analysis at 2 DIV revealed a very low amount of Gfap+ cells, while most cells resulted positive for the neuronal marker acetylated tubulin. This suggested that our primary cultures were enriched in neurons.

**Fig. 2:**
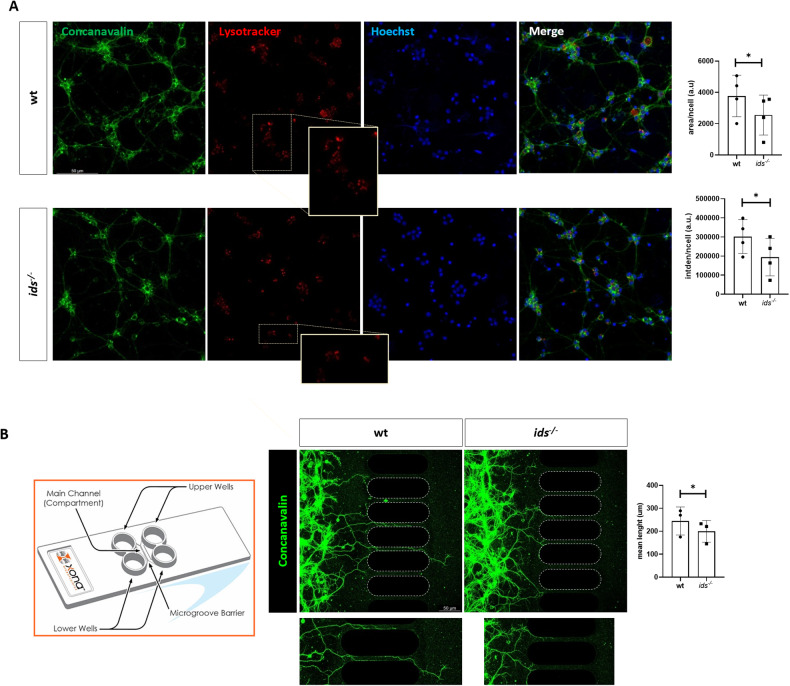
*ids* mutant fish-derived primary neurons show short axons and impaired lysosomal acidification. **A** Lysotracker staining (red) on wild-type and *ids* mutant fish-derived primary cell cultures at 2 DIV. Concanavalin (green) and Hoechst staining (blue) are reported to visualize cell membranes and nuclei, respectively. Magnifications of the Lysotracker-stained cells with reduced signal in mutant fish-derived cells are shown. On the right, bar graphs represent the area and integrated density quantifications normalized to cell number. In both cases, a significant decrease is measured in mutant fish-derived primary neurons when compared to controls (*n* = 4 independent experiments). **B** On the left, a scheme of the exploited microfluidic device is depicted. It is constituted by four wells and two main compartments divided by a microgroove barrier. Cells are seeded in one chamber and axons extend towards the other one. On the right, representative confocal images of wild-type and *ids* mutant fish-derived primary neurons seeded in the microfluidic device (2 DIV). Cultures were stained with concanavalin to easily trace processes. Below, magnifications of crossing axons are shown. The graph on the right resumes the results obtained by measuring crossing axons lengths (*n* = 3 independent experiments). *ids* mutant fish-derived neurons presented consistently shorter axons when compared to controls at 2 DIV. Scale bar: 50 μm. Data are expressed as the mean ± SD (*p < 0.05; *t*-test).

Then we analysed the extent of lysosomal acidification by Lysotracker staining since acidification constitutes one of the most important features of functional lysosomes. When looking at 2 DIV primary neurons, we found a significant decrease in Lysotracker-positive area and intensity (integrated density) in *ids* mutant conditions with respect to controls (Fig. 2A). This could suggest either a reduction in the total number of lysosomes possibly due to lysosomal biogenesis impairment or a defect in proper organelle acidification.

**Fig. 3:**
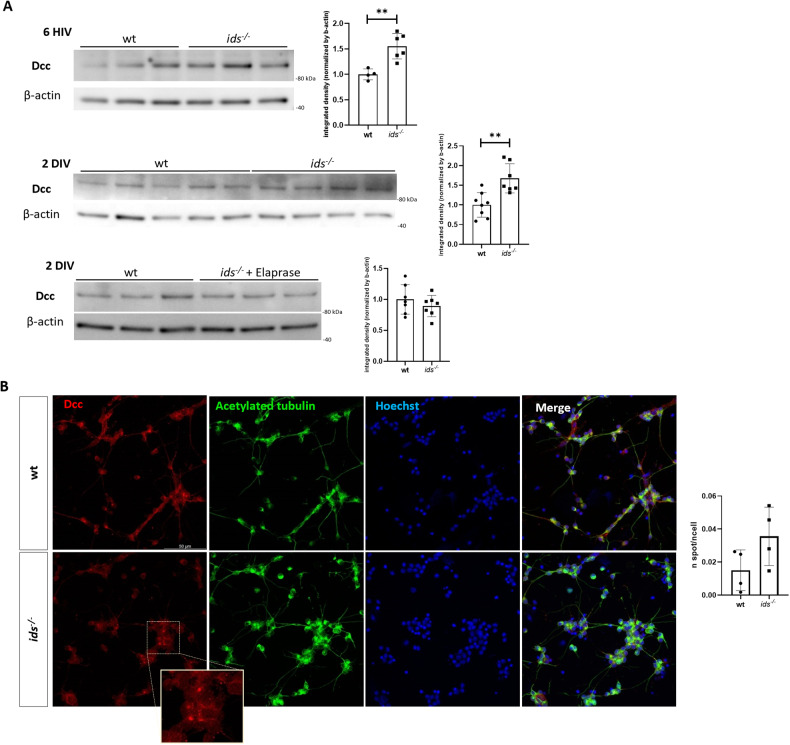
*ids* mutant fish-derived primary neurons exhibit Dcc upregulation with enrichment of Dcc-positive puncta. **A** Significant Dcc upregulation in *ids* mutant fish-derived primary neurons was detectable since 6 HIV (at least *n* = 4 biological replicates) and maintained at 2 DIV (*n* = 7 biological replicates), as depicted by western blots and related bar graphs. IDS recombinant enzyme (*Elaprase*) treatment of *ids* mutant-derived cells restores Dcc levels to those of wild-type cells (*n* = 7 biological replicates). **B** Representative Dcc immunofluorescence on wild-type and *ids* mutant fish-derived primary cells at 2 DIV. Acetylated tubulin (green) was used to trace neuronal processes. The bar graph on the right displays the quantification of Dcc-positive aggregates per cell. The magnification highlights Dcc-positive spots (*n* = 4 independent experiments). Scale bar: 50 μm. Data are expressed as the mean ± SD (**p < 0.005; *t*-test).

Subsequently, we proceeded with a phenotypical characterization of primary neurons by evaluating axonal growth using a microfluidic device. This approach allowed us to trace axonal elongation given the physical separation between cell bodies and processes. At 2 DIV, *ids* mutant-derived neurons exhibited shorter processes when compared to control ones, pointing out abnormal axon extension in basal conditions (Fig. 2B). Starting from this evidence, we performed a time course analysis of Dcc protein levels. *ids* mutant-derived primary neurons showed significant Dcc upregulation with respect to wild-type already at 6 hours in vitro (HIV), with the upregulation still traceable at 2 DIV (Fig. 3A), supporting the observations made on dissected heads and brains. Moreover, to spatially describe Dcc patterning, we performed immunofluorescence analysis on fixed primary neurons at 2 DIV: as depicted in Fig. 3B, a dotted Dcc-positive signal was detected in immunostained samples; moreover, when looking at immunolabeled spots, we found a slight increase in the number of Dcc-positive puncta in mutant conditions with respect to control. To confirm that Dcc upregulation occurred as a consequence of *ids* loss of function, we treated *ids* mutant primary neurons with *Elaprase*, the human recombinant IDS enzyme: consistently, *Elaprase* was able to rescue Dcc protein levels at 2 DIV (Fig. 3A).

In conclusion, we found that MPS II primary neurons exhibited defective axonal elongation and the previously detected Dcc upregulation could be mainly related to the neuronal population.

### Dcc is detected in P62-enriched cytosolic fraction in *ids* mutant larvae

Dcc, as well as many other transmembrane receptors, experiences an intense trafficking between cytoplasmic (vesicular) and membrane compartments. These different localizations allow to restrict or hamper the activation of the downstream signalling pathway, resulting in the precise regulation of growth cone dynamics [20, 21].

**Fig. 4:**
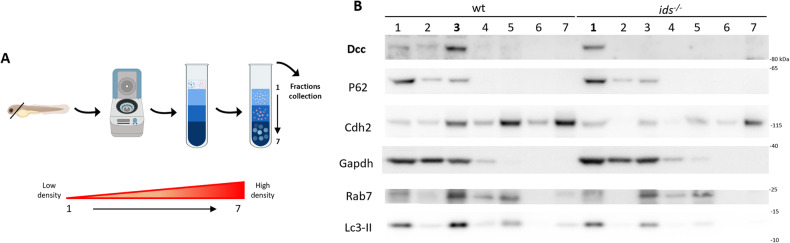
P62 protein levels are increased in Dcc-enriched fractions of *ids* mutant fish-derived protein lysates. **A** Graphical representation of the workflow adopted for the fractional precipitation assay (created with Biorender). Briefly, 2 dpf dissected heads were homogenized and subjected to first centrifugation; then, the supernatant was loaded on top of a sucrose column to be submitted to ultracentrifugation. Finally, fractions were retrieved from the top (low density) to the bottom (high density) of the sucrose gradient. **B** Representative western blot depicting wild-type and *ids* mutant fish lysates subjected to fractional precipitations. Fractions are reported from 1 to 7, with bold numbers highlighting the fraction with higher Dcc enrichment (fraction 3 for control and fraction 1 for *ids* mutant-derived lysates). The distributions of P62, N-cadherin (Cdh2), Gapdh (glyceraldehyde-3-phosphate dehydrogenase), Rab7 (Ras-associated binding protein 7), and Lc3-II were analysed. While the majority of markers were distributed in the same way in the two genotypes, P62 was particularly enriched in the same fraction displaying high Dcc protein levels only in *ids* mutant conditions.

Given these premises, we verified whether Dcc might show a differential subcellular localization in *ids* mutant and wild-type conditions. To this purpose, we set up an optimized protocol for fractional precipitation starting from 2 dpf zebrafish dissected heads. Once assessed the reproducibility of the protocol and thus the possibility of obtaining membrane-, cytosolic- and vesicular-enriched fractions (Supplementary Fig. 2A), we applied the pipeline to wild-type and *ids* mutant fish-derived protein lysates. By sucrose gradient separation, we retrieved different fractions from lower to higher densities, progressively numbered (1–7) and then analysed by western blot (Fig. 4A). When looking at Dcc, it differentially distributed in wild-type and *ids* mutant fish-derived lysate fractions. While in control conditions, most of Dcc could be found in fraction 3 (herein referred to as *wt3*), in *ids* mutant, it was detected only in fraction 1 (referred to as *ids1*) (Fig. 4B). Neither *wt3* nor *ids1* showed N-cadherin (Cdh2)/Rab7 enrichment, underlying the fact that plasma membrane, as well as endosomal fractions, were depleted of Dcc. On the other hand, autophagic marker Lc3-II (microtubule-associated protein 1 A/1B-light chain 3) displayed relatively high levels in both *wt3* and *ids1*. The marker that showed a clearly differential distribution between the two genotypes was P62 (Sequestosome-1, SQSTM): indeed, while *wt3* displayed low P62 levels, *ids1* showed very high P62 enrichment. In addition, immunofluorescence analysis on 2 DIV primary neurons showed an increasing trend in P62-Dcc co-localization in *ids* mutant conditions (Supplementary Fig. 2B).

From these observations, we could conclude that Dcc was mainly localizing within the soluble compartment, but only in *ids* mutant conditions it was highly enriched in the fraction containing higher protein levels for the autophagic receptor P62.

### Inhibition of proteasomal degradation in wild-type neurons results in Dcc upregulation, thus resembling *ids* mutant phenotype

Since in *ids* mutant conditions we detected high P62 protein levels in the Dcc-enriched fraction, we next evaluated autophagic and proteasome-mediated degradation pathways. Indeed, P62 represents a key player shared between the abovementioned cascades [22]. Moreover, autophagy and protein degradation are known to be impaired in different types of MPSs, thus underlying their tight relation with lysosomal homoeostasis [3, 23–25].

**Fig. 5:**
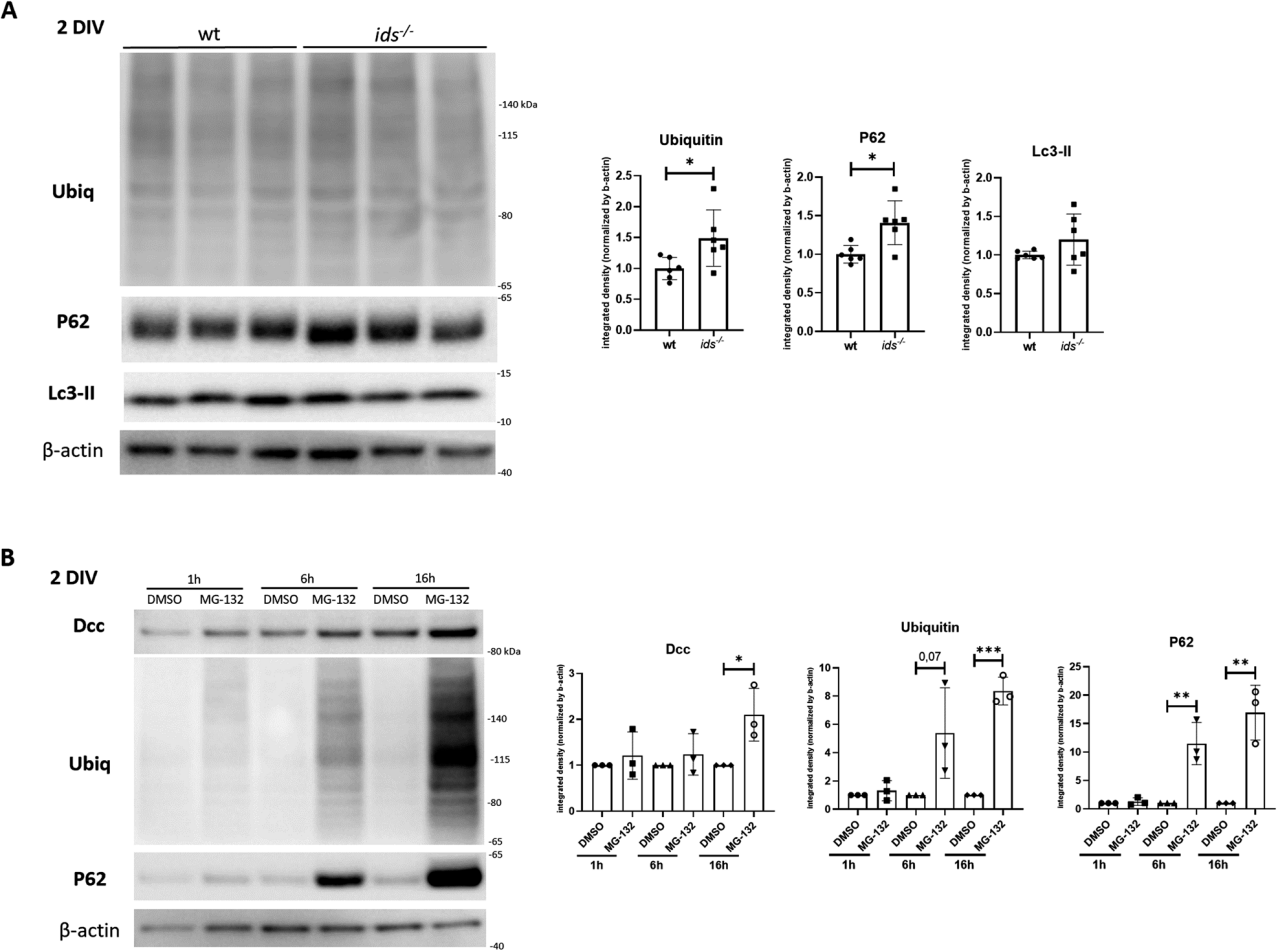
Proteasome inhibition in wild-type primary neurons results in Dcc upregulation. **A** Western blot analysis on 2 DIV wild-type and *ids* mutant fish-derived primary neurons showed significant upregulation of polyubiquitinated proteins and P62, as depicted by the bar graphs on the right. On the other hand, no differences were found for Lc3-II (*n* = 6 biological replicates). **B** Wild-type fish-derived primary neurons were treated with MG-132 (proteasome inhibitor) for 1, 6, or 16 hours and then analysed at 2 DIV. On the left, representative western blots for Dcc, ubiquitin and P62. After 16 hours of treatment, Dcc levels were significantly increased when compared to DMSO (*n* = 3 independent experiments). Data are expressed as the mean ± SD (*p < 0.05; **p < 0.005; ***p < 0.001; *t*-test).

We checked for P62, Lc3-II, and polyubiquitinated proteins in primary neurons at 2 DIV. When comparing *ids* mutant and control fish-derived cell cultures, we found significantly upregulated P62 and polyubiquitinated protein levels in *ids* mutant conditions, whereas no significant differences could be detected for Lc3-II (Fig. 5A). For this reason, we wondered whether Dcc upregulation reported in mutant fish-derived primary neurons might be attributable to defective proteasomal degradation rather than autophagic impairment. Toward this aim, we treated wild-type-derived primary neurons with the proteasomal inhibitor MG-132 and checked for Dcc protein levels. Relative levels of polyubiquitinated proteins and P62 were used in all experiments to verify treatment efficacy. While no significant differences were found at 1 and 6 hours of MG-132 treatment, after 16 h we were able to find sustained Dcc upregulation, successfully mimicking the dysregulation seen in *ids* mutant conditions (Fig. 5B). Collectively, our results supported the hypothesis that proteasomal degradation could be one of the main pathways whose deficiency led to Dcc dysregulation in MPS II primary neuronal cell cultures.

### *ids* mutant larvae exhibit alterations in the anxiety-like response without showing defects in retinotopic maps

Since Dcc-mediated signaling pathway has been recognized as fundamental for correct optic nerve fasciculation at the optic stalk [26] and MPS II patients show visual-related symptoms such as retinal degeneration and impaired stimuli processing [4], we assessed whether these phenotypes could be recapitulated in an MPS II zebrafish model. 6 dpf wild-type and *ids* mutant *Ath5 GCaMP:GFP* zebrafish larvae were subjected to whole-brain recording and neuronal activation was traced in response to looming and moving stimuli. Even if control larvae showed higher variability when compared to mutant ones, no significant differences were evident between the two genotypes, neither in retinotopic mapping (i.e. the position flaring regions triggered by specific visual stimuli inside the optic tectum) (Supplementary Fig. 3A) nor in GFP signal intensity (Supplementary Fig. 3B).

**Fig. 6:**
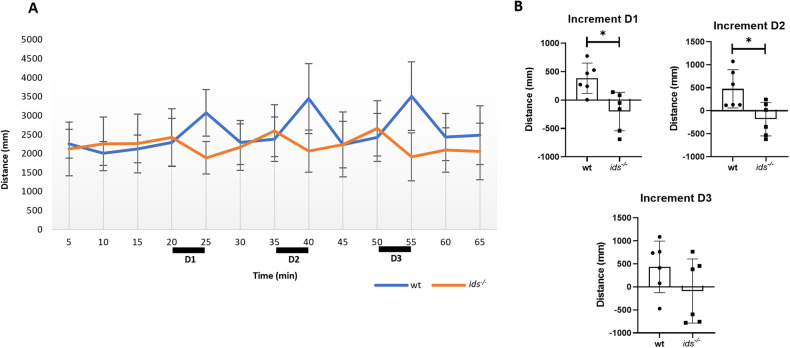
*ids* mutant larvae exhibit significantly altered anxiety-induced response. **A** 15 dpf *ids* mutant and wild-type larvae were challenged with the LDT test to elicit an anxiety-like response. The bar graph on the left reports the mean distance run by each group of larvae every five minutes (representative trial). Black boxes are put in correspondence to the five minutes of complete darkness (D1: first dark phase, 20–25 min; D2: second dark phase, 35–40 min; D3: third dark phase, 50–55 min). Data are expressed as the mean ± SD (*n* = 6 biological replicates). **B** The bar graphs depict the evaluation of the “increments” for each dark phase. During D1 and D2 *ids* mutant larvae display a negative value of the increment, whereas controls increment their locomotion, as expected. In D3, no differences were observed between genotypes (*n* = 6 independent experiments). Data are expressed as the mean ± SD (*p < 0.05; *t*-test).

Then, to evaluate larval response toward a stressing stimulus, we applied the light/dark transition (LDT) test. In physiological conditions, larvae subjected to sudden darkness increase their locomotion due to their natural preference for light environments [27]. For this reason, the LDT test has been widely used to assess the anxiety-induced response [28]. As MPS II neuronopathic patients frequently suffer from anxiety [29] and the anxious phenotype has been previously associated with defects in axon guidance molecules [30], we compared *ids* mutant and wild-type larvae for anxiety-like responses. In particular, we evaluated the locomotion of 15 dpf larvae since, at this time point, fish behaviour exhibits a higher degree of complexity with respect to the first week of age. Our results showed that, while wild-type larvae displayed the stereotypic hyperactivity induced by the dark phase, *ids* mutant fish significantly decreased their motility (Fig. 6A). This resulted evident when calculating the “increment”, i.e. the difference between the value of distance travelled during the dark phase, and the value of the distance travelled in the previous 5 minutes of light. In the first and second dark phases, *ids* mutant larvae showed a completely opposite phenotype when compared to controls, while no differences were reported for the third dark period, suggesting the instauration of habituation towards the stimulus (Fig. 6B).

Overall, our data suggested that, even if no difference could be found in retinotopic mapping, *ids* mutants displayed a profoundly altered phenotype when challenged with an anxiety-inducing stimulus, thus pointing out possible alterations in stimuli processing.

## Discussion

Iduronate-2-sulfatase (IDS) is a lysosomal enzyme whose mutations are associated with the onset of Hunter syndrome (Mucopolysaccharidosis type II), a rare X-linked lysosomal storage disorder (LSD). As the name suggests, LSD aetiology has been traditionally ascribed to the lysosomal engorgement caused by the accumulation of undegraded substrates. However, increasing evidence pointed out that lysosomal homoeostasis is tightly interconnected with several intracellular and extracellular events such as vesicle trafficking, autophagy and signalling pathway regulation, thus challenging the view of lysosomes as a merely catabolic compartment [1, 2]. This novel perspective was further supported by our previous findings on *ids* morphants and stable *ids* mutant zebrafish, which displayed signalling pathways alterations (FGF, Shh, Wnt) since early developmental stages [16, 31].

The fact that neuronopathic MPS II patients usually develop CNS abnormalities from early childhood highlights the importance of lysosomal function in CNS development. In particular, it is widely documented that lysosomes play a crucial role in neuronal differentiation and maintenance [32–34]. In this context, an RNA-seq analysis performed on an MPS II mouse brain revealed dysregulation of a great number of pathways, including those involving synapse maturation, calcium signalling and axon guidance [8]. In line with this evidence, in this work, we demonstrate that the axon guidance receptor Dcc is significantly dysregulated in the brain of MPS II fish since the earliest stages of neurodevelopment. A quantitative increase of Dcc protein levels was consistently found both in whole dissected heads and brains as well as in isolated neurons from *ids* mutant fish. In the latter case, the establishment of neuronal-enriched primary cultures allowed us to precisely track the molecular defects upstream of Dcc dysregulation. First, *ids* mutant neurons showed reduced acidified lysosomes. Interestingly, we recently reported the same aberrant phenotype in differentiated IDS mutant human neuronal cells [35], underlying that this defect is maintained across species harbouring an IDS defective activity. In addition, we showed a significant upregulation of both polyubiquitinated proteins and P62 in primary neurons obtained from 2 dpf *ids* mutant brains. While the correlation between lysosomal storage disorders and the accumulation of polyubiquitinated substrates has been largely described [36–39], this is the first evidence of polyubiquitinated substrate accumulation in the early life stages of a lysosomal storage disorder experimental model. Notably, Bifsha and colleagues reported a significant downregulation of the major ubiquitin C-terminal hydrolase UCH-L1 in the brain of a 3 months-old mouse model of Sandhoff disease (an LSD), together with increasing levels of polyubiquitated aggregates [40].

Moreover, our data indicated that Dcc was highly concentrated in the P62-enriched fraction of *ids* mutant fish-derived lysates. Given the concomitant accumulation of polyubiquitinated substrates in neuronal-enriched cultures, we suspected that the increase in Dcc protein levels could be due to proteasomal degradation defects. As a further hint, no significant differences in Lc3-II protein levels were found between *ids* mutant and control primary neurons, suggesting that autophagic impairment might not be the leading cause of Dcc dysregulation, at least at early developmental stages. Interestingly, an MG-132-based inhibition of proteasomal degradation in control neurons led to Dcc upregulation, thus resembling the phenotype of *ids* mutant cells and confirming our working hypothesis. Moreover, our results perfectly agree with what has been reported concerning Dcc degradation pathways. In particular, Kim and colleagues demonstrated that embryonic mouse cortical neurons treated with MG-132 displayed Dcc upregulation [14].

Finally, behavioural analyses of *ids* mutant and age-matched control fish revealed opposite responses towards an anxiety-like stimulus, with mutant fish exhibiting reduced overall motility when subjected to complete darkness. Notably, it has been proposed that zebrafish behaviour depends on the magnitude of anxiety status: larvae with moderate/low anxiety levels respond to a stressing stimulus with the stereotyped escape phenotype, thus increasing their swimming. On the other hand, fish with high anxiety levels freeze rather than escape [41]. This might suggest that already at larval stages MPS II fish exhibited higher anxiety distress when compared to controls, reflecting a characteristic MPS II behavioural trait. Moreover, it has been extensively shown that alterations in Dcc protein levels impact on anxiety-like behaviours and depression [42–44]. Therefore, we could speculate that MPS II larval anxiety behaviour might be, at least in part, also due to Dcc imbalance.

In conclusion, in this work we shed light on an early axon guidance defect due to *ids* loss of function. We found that Dcc upregulation could be due to its defective proteasomal degradation, supporting the hypothesis that lysosomal defects impinge on receptor recycling and internalization [39].

Our research aligns with the idea that understanding the earliest molecular changes associated with Mucopolysaccharidosis type II is of paramount importance for prompt therapeutic interventions and the development of new therapeutic strategies.

## Materials and methods

### Zebrafish lines

#### ids mutant zebrafish line (ids^ia200/ia200^)

The *ids* mutant zebrafish line was previously established by Bellesso and colleagues [16]. Briefly, the mutant harbours a 5 base pair deletion within *ids* exon2 (chr14), causing a frameshift in the reading frame and the generation of a premature stop at codon 118; this leads to the translation of a non-functional Ids protein.

Animals ranging from 24 hpf to 15 dpf were analysed regardless of sex, which is determined at 2–3 months of age in the zebrafish model. Ethical procedures with fish were approved by the Italian Ministry of Health and the Local Institutional Review Board of University of Padova (OPBA) (code 60/2019).

#### Ath5-GCaMP6s

*Ath5-GCaMP6s* larvae were used for tectal neuropil imaging. This transgenic line was kept in Tupfel long fin nacre (TLN) background. 6 dpf larvae were used for experimental procedures.

### Protein extraction and western blot

Proteins were extracted from zebrafish dissected heads, dissected brains or primary cells. Samples were placed in Tissue Extraction Reagent (Thermo Fisher, Monza, Italy) added with protease inhibitors (Thermo Fisher, Monza, Italy) and phosphatase Inhibitors (ThermoFisher, Monza, Italy) to prevent protein degradation. Samples were next manually homogenized, subjected to sonication and then centrifuged at 16,000 × *g* for 30 min at 4 °C. The supernatant was collected and protein concentration was determined by the BCA Protein Assay Kit (Thermo Fisher, Monza, Italy). Sample absorbance was measured at 540 nm by a spectrophotometric analysis.

Extracted proteins were analysed by SDS–PAGE. Protein lysates (10 μg for tissues-derived samples and 15 μg for in vitro extracts) were supplemented with a 1X SDS sample buffer (Thermo Fisher, Monza, Italy) containing 5% 2-Mercaptoethanol (Merck, Milano, Italy), heated at 75 °C for 10 min and run in precast acrylamide gels (4-12% Bis–Tris SDS–PAGE (Thermo Fisher, Monza, Italy) in MOPS running buffer (Thermo Fisher, Monza, Italy). Proteins were transferred on PVDF membranes (Thermo Fisher, Monza, Italy) in 25 mM Tris, 192 mM glycine, and 20% methanol (v/v) using the standard “sandwich” assembly and incubated with primary antibodies overnight at 4 °C. Primary antibodies were used at 1:1000 final dilution (see Table 1). After washes in TBST (Tris-buffered saline-0.1%Tween 20), incubation with secondary horseradish peroxidase (HRP)-conjugated antibody was performed at room temperature (RT) for 1 h at 1:2000 final dilution (see Table 2). Chemiluminescent signals were detected incubating the blotted membranes with the Supersignal West Pico Chemioluminescent substrate kit (Thermo Fisher, Monza, Italy) and visualized by Image Quant Las 4000 (GE Healthcare, Milano, Italy) and analysed by ImageJ software (https://imagej.nih.gov/ij/). When needed, membranes were stripped using a commercially available solution (Restore PLUS Western blot stripping buffer (Thermo Fisher, Monza, Italy). For uncropped original western blot see Supplementary Information.

**Table 1:** Western Blot primary antibodies.

Target	Code	Host
Dcc	Antibodies Online (Aachen Germany), ABIN2782671	Rabbit
Cdh2	Genetex (lton Pkwy Irvine, CA, USA), GTX125885	Rabbit
P62	Cell Signaling (Milano, Italy), #5114	Rabbit
Rab7	Abcam (Cambridge, UK), ab50533	Mouse
Lc3-II	Thermo Fisher (Monza, Italy), PA1-16930	Rabbit
β-actin	Santa Cruz (Dallas, TX, USA), sc-56459	Mouse
Gapdh	Abcam (Cambridge, UK), ab9485	Rabbit
Ubiquitin	Cell Signaling (Milano, Italy), #3936	Mouse

**Table 2:** Western blot secondary antibodies (HRP-conjugated).

Antibody	Code
Anti- Mouse	Fortis Life Science (Waltham, MA USA), A90-116P
Anti-Rabbit	Jackson ImmunoResearch (Ely, UK), 111-035-019

### Whole-mount immunofluorescence on dissected brains

Whole-mount immunofluorescence was performed on 4% PFA fixed dissected brains. Samples were incubated in Tris–HCl 150 mM pH 9 for 5 min and then placed at 70 °C for 20 min. Brains were then rinsed in PBS Triton (PBS+TritonX-100 0.25% Thermo Fisher, Monza, Italy). Saturation was performed with 1% BSA, 2% sheep serum, 1% DMSO in PBS Triton 0.25%.

Primary and secondary antibodies were used at 1:100 final dilution with O/N incubations (see Tables 3 and 4). Labelled brains were then mounted on 1.5% low melting agarose for confocal imaging.

**Table 3:** Immunofluorescence primary antibodies.

Target	Code	Host
Dcc	Antibodies Online (Aachen Germany), ABIN2782671	Rabbit
Acetylated tubulin	Merck (Milano, Italy), T7451	Mouse
Gfap	Abcam (Cambridge, UK), ab154474	Mouse
P62	Progen (Heidelberg, Germany), GP62-C	Guinea Pig

**Table 4:** Immunofluorescence secondary antibodies (fluorophore-conjugated).

Antibody	Code
Anti-Mouse FITC	Thermo Fisher (Milan, Italy), F2761
Anti-Rabbit TRITC	Thermo Fisher (Milan, Italy), A16101
Anti-Guinea Pig FITC	Jackson ImmunoResearch (Ely, UK), 106-095-003

### Zebrafish-derived primary neuronal cell cultures

Brains were dissected from 2 dpf bleached larvae as previously described [45]. Brain pools were collected in Hank balanced salt solution (HBSS) supplied with penicillin/streptomycin (Thermo Fisher, Monza, Italy) at RT. Cell dissociation was performed by 5 mg/ml Collagenase II (Thermo Fisher, Monza, Italy) incubation for 20 min at 37 °C. Isolated cells were centrifuged at 800 × *g* for 5 min, and the pellet was resuspended in a culture medium. Complete medium was composed by DMEM/F-12, HEPES (Thermo Fisher, Monza, Italy) supplemented with 2% penicillin/streptomycin-glutamine (Thermo Fisher, Monza, Italy), 1% N2 supplement (Thermo Fisher, Monza, Italy), 2% B-27 supplement (Thermo Fisher, Monza, Italy), 0.25 μg/ml Amphotericin B (Thermo Fisher, Monza, Italy), 0.1% BSA (Merck, Milano, Italy), 2 mM Glutamine (Thermo Fisher, Monza, Italy) and 0.5 μg/ml Rifampicin (Merck, Milano, Italy). Approximately 400,000 cells were seeded on poly-lysinated wells (Merck, Milano, Italy) and maintained at 28 °C. Cell medium was replaced after 1 day in vitro (DIV). Bright-field images were taken using a Leica DMIL LED microscope, ×40 objective (Leica, Milano, Italy).

As regards treatments, *ids* mutant-derived primary cultures were treated with 15 nM *Elaprase* (Takeda, Tokyo, Japan) in complete growth medium for 2 DIV and compared to wild-type controls, with medium replacement at 1 DIV. Inhibition of proteasome activity was achieved with 20 μM MG-132 (Merck, Milano, Italy) in a complete growth medium: incubations were performed for 1, 6, and 16 h and lysates were collected at 2 DIV. Each time point was compared with the matched DMSO condition.

### Immunofluorescence and Lysotracker staining on primary cell culture

For immunofluorescence assays, primary cell cultures were fixed with 2% PFA supplied with 1 mM CaCl_2_, 1 mM MgCl_2_ and 4% sucrose for 10 min RT. After rinsing twice with PBS, blocking was performed with 10% sheep serum in PBS for 1 h at RT. Primary antibodies were incubated O/N at 4 °C (see Table 3). After two washes with PBS, cells were incubated with a fluorescent secondary antibody (see Table 4) supplemented with 5.5 μM Hoescht (Thermo Fisher, Monza, Italy) at RT for 1 h and finally washed with PBS.

For Lysotracker staining, primary cultures were incubated with Lysotracker Red 500 nM (Thermo Fisher, Monza, Italy) for 1 h at 28 °C and post-fixed according to the abovementioned protocol. After one wash in PBS, cells were incubated with 0,1 mg/ml concanavalin (Thermo Fisher, Monza, Italy) 5,5 μM Hoechst (Thermo Fisher, Monza, Italy) for 10 minutes at RT to label whole cell membranes and nuclei, respectively. All samples were mounted with Fluoromount (Thermo Fisher, Monza, Italy) and processed by confocal microscopy.

### Microfluidic assays

Microfluidic chambers (Xona Microfluidics, XonaChip 150 μm, Research Triangle, NC, USA) were coated with poly-lysine (Merck, Milano, Italy) according to manufacturer’s guidelines. Approximately 200,000 cells were seeded on one side of the device; during the seeding phase, 20 μl of cell medium (composition abovementioned) was added to the opposite chamber to restrain the cells inside the seeding compartment, thus preventing microgrooves crossing. A 20 μl volume difference was set between the seeded and empty chambers; since this difference could be maintained up to 24 h, it needed to be re-established at 1 DIV by fresh medium addition. Cultures were then maintained at 28 °C for 2 DIV. For cell membranes visualization, cells were incubated with 0.1 mg/ml concanavalin (Thermo Fisher, Monza, Italy) for 10 min, rinsed with PBS and acquired by confocal microscopy.

### Confocal analysis

Whole brains were analysed by confocal microscopy using a Nikon C2 H600L confocal microscope (Milan, Italy), ×40 immersion objective with Z-stack of ~3 μm (imaging depth ~200 μm). For 3D reconstructions, NIS-Elements Viewer (Nikon, Firenze, Italy) software was used.

Fluorescent images of primary cell culture and microfluidic device were acquired using Leica Stellaris confocal microscope equipped with a charge-coupled device camera and processed with the Leica LASX software (Leica, Milano, Italy). Each field was acquired with a Z-stack of 1 μm using a ×63 immersion objective (cell culture immunofluorescence) or 0.5 μm, ×40 immersion objective, ×0.75 zoom (microfluidic devices). For each coverslip, at least six or seven fields were acquired. Confocal images were processed using ImageJ software (https://imagej.nih.gov): thresholded area and the integrated density of the total sum of slices per field were used to compare the different genotypes. Pearson’s coefficient was calculated using the JaCoP plugin for the co-localization between P62/Dcc.

### Separation of subcellular fractions: fractional precipitation

Fractional precipitation was performed on 2 dpf head lysates: dissected heads were collected in PBS supplemented with PMSF 1x and then manually homogenized (in a glass potter and with a glass pestel) in Tris 1 mM, EDTA 2 mM pH 7.4 buffer supplemented with protease and phosphatase inhibitors (Thermo Fisher, Monza, Italy). Head extracts were subjected to a 3 min 800 × *g* centrifugation and surnatants, at the final concentration of 1.5 mg/ml, were loaded on the top of a sucrose gradient (5–20–38% sucrose in Tris 1 m M, EDTA 2 mM pH 7,4). Subsequent ultracentrifugation (Beckman Optima L-90K Ultracentrifuge, Milano, Italy) was performed with SW 60 Ti Swinging-Bucket Rotor (Beckman Coulter, Milano, Italy) at 40,000 rpm for 1 h at 4 °C, slow acceleration and no brake. Fractions were recovered manually from the top to the bottom of the column and then concentrated by exploiting the trichloroacetic acid (TCA)–Na-deoxycholate (DOC) method and speed vacuum (Savant SVC 100H Speed Vac Concentrator, Thermo Fisher, Monza, Italy). Pellets were resuspended in 1x SDS sample buffer (Thermo Fisher, Monza, Italy) and analysed by western blot as previously described.

### Retinotopic mapping

For retinotopic mapping, 6 dpf *Ath5 GCaMP:GFP* wt and *ids* mutant larvae were placed in a 2% low melting agarose drop and analysed by two-photon microscopy with a ×16 objective (Thorlabs, Newton, NJ, USA). Larvae were placed at 90° with respect to a 3 cm far display on which stimuli were projected. In particular, both looming and moving stimuli, generated in Python using Stytra [46], were presented monocularly. Controlateral retinal ganglion cell’s tectal projections were recorded by whole-brain scanning (volume covered 100 × 100 × 55 μm^3^) and analysed through a custom Python script. Basically, each pixel track was compared with the stimulation track in time; this led to the definition of *coefficients* and *scores*. The *coefficient* indicated was correlated to pixel intensity for a given stimulation (higher coefficients corresponded to higher response intensity), while with *scores*, we referred to pixel response variability (higher scores corresponded to higher reliability in the analysis).

### Light/dark transition test

For the assay, 15 dpf fish were placed in a 12-well plate and tested with Danio vision (Noldus, Wageningen, Netherlands). The design of the test has been adapted from a light–dark challenge protocol developed for 5 dpf larvae [28]. The trial consisted of an initial acclimation period of 20 min to give fish the time to adjust to the new environment. This period was followed by a dark phase of 5 min and another light phase of 10 min, repeated 3 times. This results in six alternating dark and light phases. Data were obtained by Noldus software EthoVision XT (version 14, Wageningen, Netherlands) by setting the minimal distance moved (mdm) filter at 0.5 mm to reduce background noise.

### Statistical analysis

Results were expressed as mean ± SD (standard deviation). Data were analysed according to the assay using a two-tailed unpaired/paired Student’s *t*-test for comparison. *p* values < 0.05 were considered statistically significant. The confidence level for significance was 95%. Data were analysed using Prism software version 8 XLM (GraphPad Software). Additional statistical details can be found in the figure legends.

### Supplementary information

Supplementary material Manzoli et al., 2024

## Data Availability

All relevant data supporting the key findings of this study are reported within the article and its Supplementary Information. Additional information will be provided upon request to Authors.
