# Supplementary material for: Mucopolysaccharidosis type II zebrafish model exhibits early impaired proteasomal-mediated degradation of the axon guidance receptor Dcc
Source: Cell Death Dis. 2024 Apr 16;15(4):269. doi: 10.1038/s41419-024-06661-2 (PMC11021486; doi:10.1038/s41419-024-06661-2)

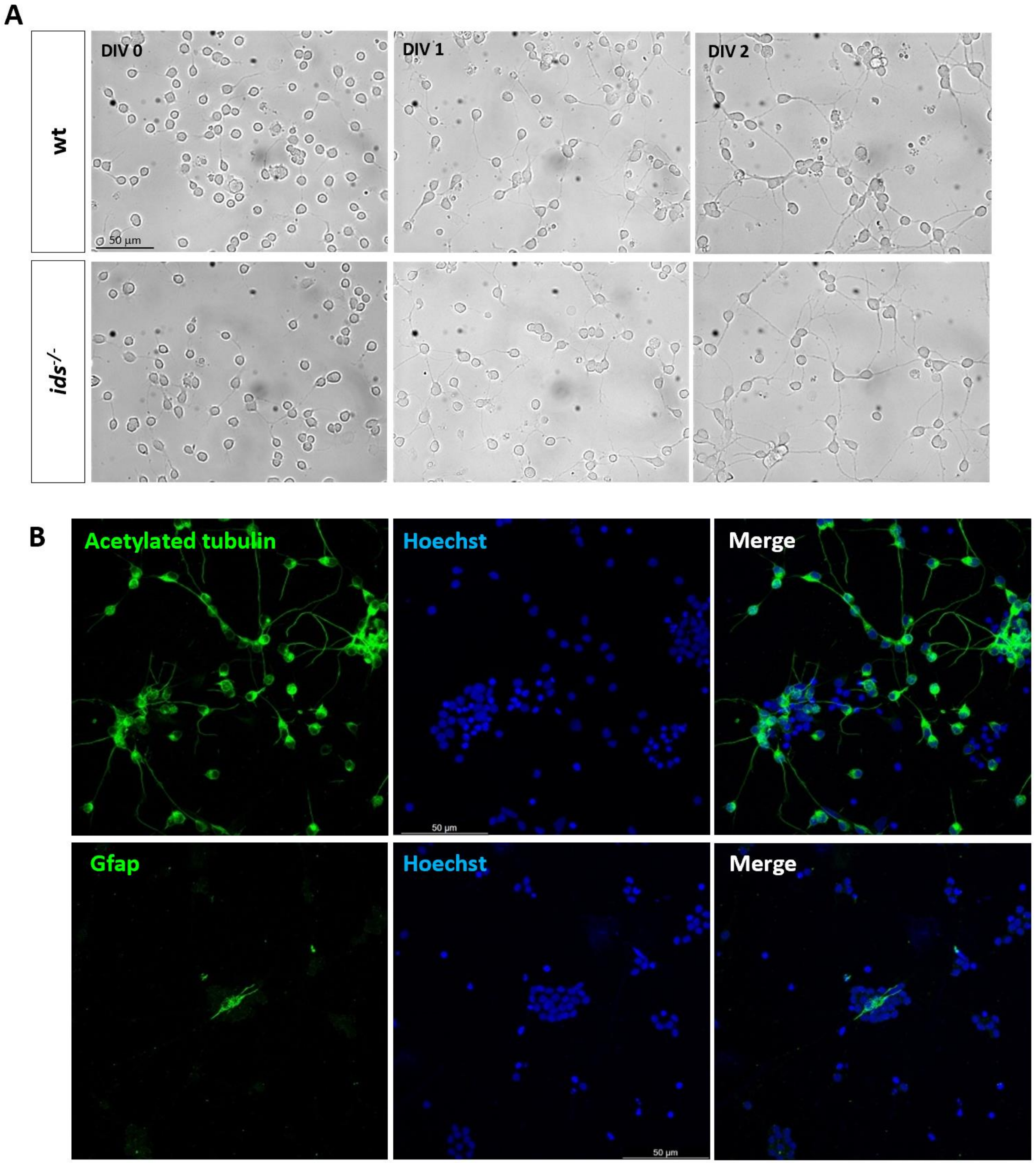
A
DIV 0
DIV 1
DIV 2
wt
ids-/-
50 µm
B
Acetylated tubulin
Hoechst
Merge
Gfap
Hoechst
Merge
50 µm
50 µm

Supplementary Fig. 1: Generation of neuronal-enriched primary cell cultures from 2 dpf zebrafish brain. A Bright field images of wild type and mutant fish-derived primary cell cultures from 0 to 2 days in vitro. First processes are evident already after few minutes *in vitro*. **B** On the top, representative Acetylated Tubulin/Hoechst immunofluorescence on a wild type fish-derived primary cell culture at 2 DIV. On the second raw, representative Gfap/Hoechst immunofluorescence on wild type fish-derived primary cell culture at 2 DIV. As evident from the image, the percentage of Gfap-positive cells is very low when compared to total nuclei number. Scale bar: 50 µm.

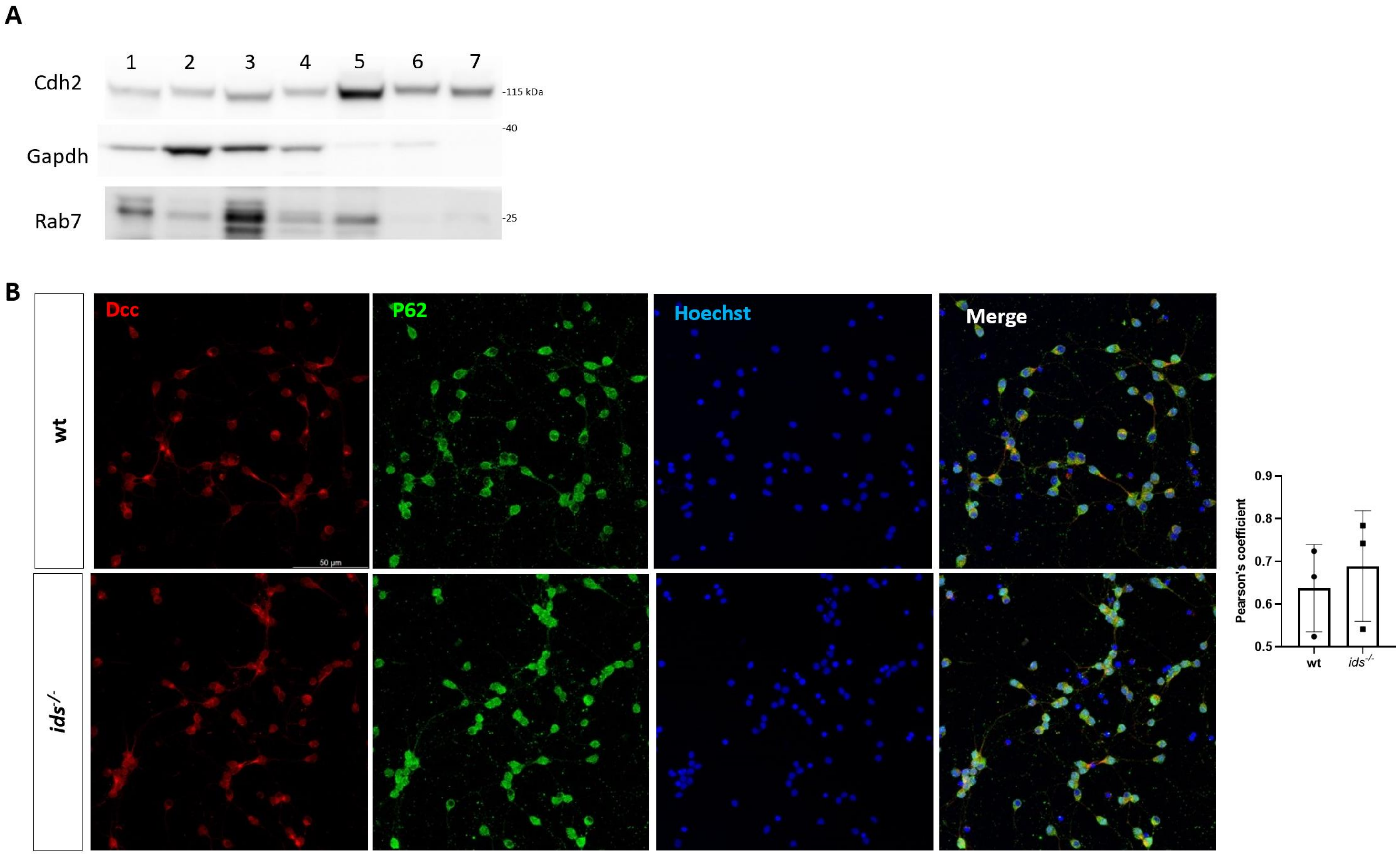
A
1
2
3
4
5
6
7
Cdh2
-115 kDa
-40
Gapdh
Rab7
-25
B
Dcc
P62
Hoechst
Merge
wt
ids-/-
50 µm
Pearson's coefficient
0.9
0.8
0.7
0.6
0.5
wt
ids-/-

Supplementary Fig. 2: Fractional precipitation, P62-Dcc immunofluorescence and co-localization analysis on primary neuronal cell cultures. A Representative western blot showing successful fractionation of wild type fish-derived head protein lysates. Cdh2 (plasma membrane), Gapdh (cystosol) and Rab7 (late endosomes) proteins are enriched in different fractions. Fractions were collected from the top (1) to the bottom (7) of the column. **B** Representative immunofluorescence for Dcc (red) and P62 (green) on 2 DIV primary neuronal cell cultures (n= 3 independent experiments). The graph reports the results of Dcc-P62 colocalization analysis (Pearson's coefficient). Scale bar: 50 µm. Data are expressed as the mean ± SD of three independent assays (t-test).

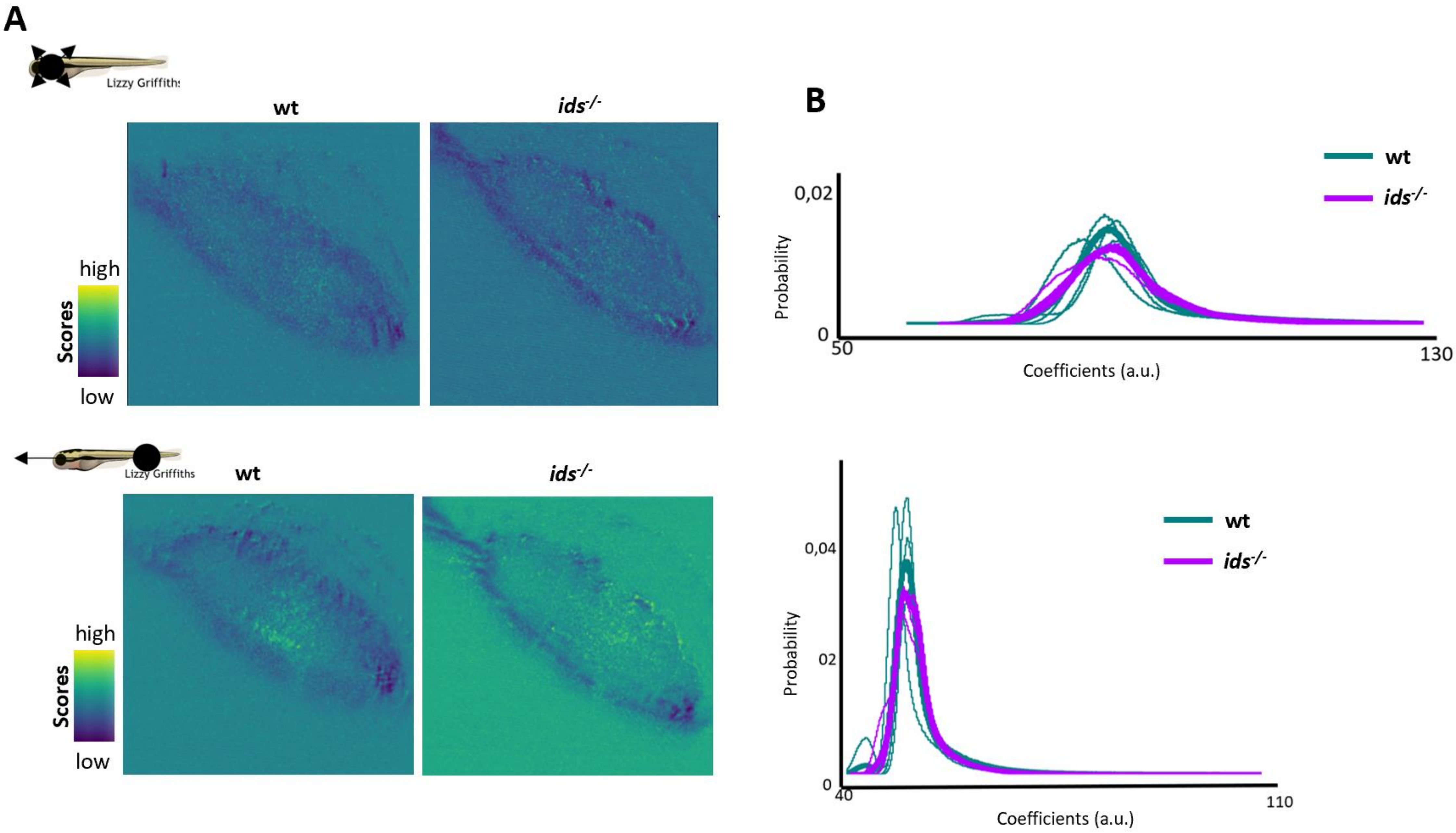

A
Lizzy Griffiths
wt
ids-/-
high
Scores
low
B
wt
ids-/-
0,02
Probability
0
50
130
Coefficients (a.u.)
Lizzy Griffiths
wt
ids-/-
high
Scores
low
wt
ids-/-
0,04
Probability
02
0
40
110
Coefficients (a.u.)

Supplementary Fig. 3: Analysis of retinotopic mapping in 6 dpf larvae through two-photon confocal imaging. A Reconstruction of retinotopic mapping of *Ath5 GCaMP:GFP* control and *ids* mutant zebrafish larvae at 6 dpf subjected to visual stimulation. The type of stimulation (looming or moving dot) is reported in the scheme on the left. Regions with higher scores are depicted in yellow while low scores pixels are shown as blue. **B** Graphical representation of coefficient related to looming and moving dot-induced responses. Each thin curve represents one single fish while bold ones represent the medium response. The curves graphically describe the distribution of arbitrary intensity values (coefficient) in the retinotopic map of wild type and mutant larvae subjected to visual stimulation. *ids* mutant and wild type fish-related curves almost coincide (at least n= 3 different subjects).

Fig. 1A

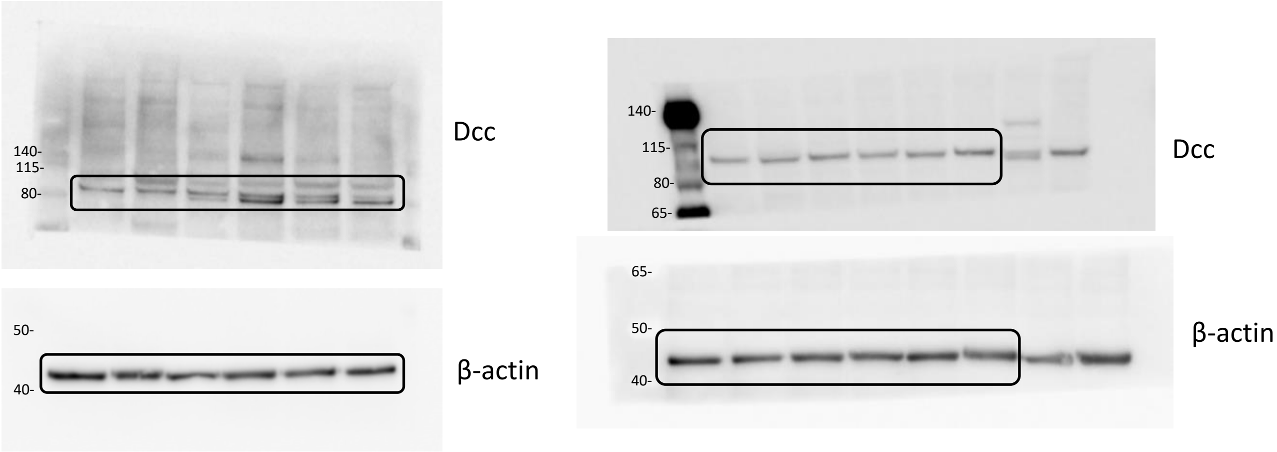
140-
115-
80-
Dcc
50-
40-
β-actin
140-
115-
80-
65-
Dcc
65-
50-
40-
β-actin

Fig. 1B

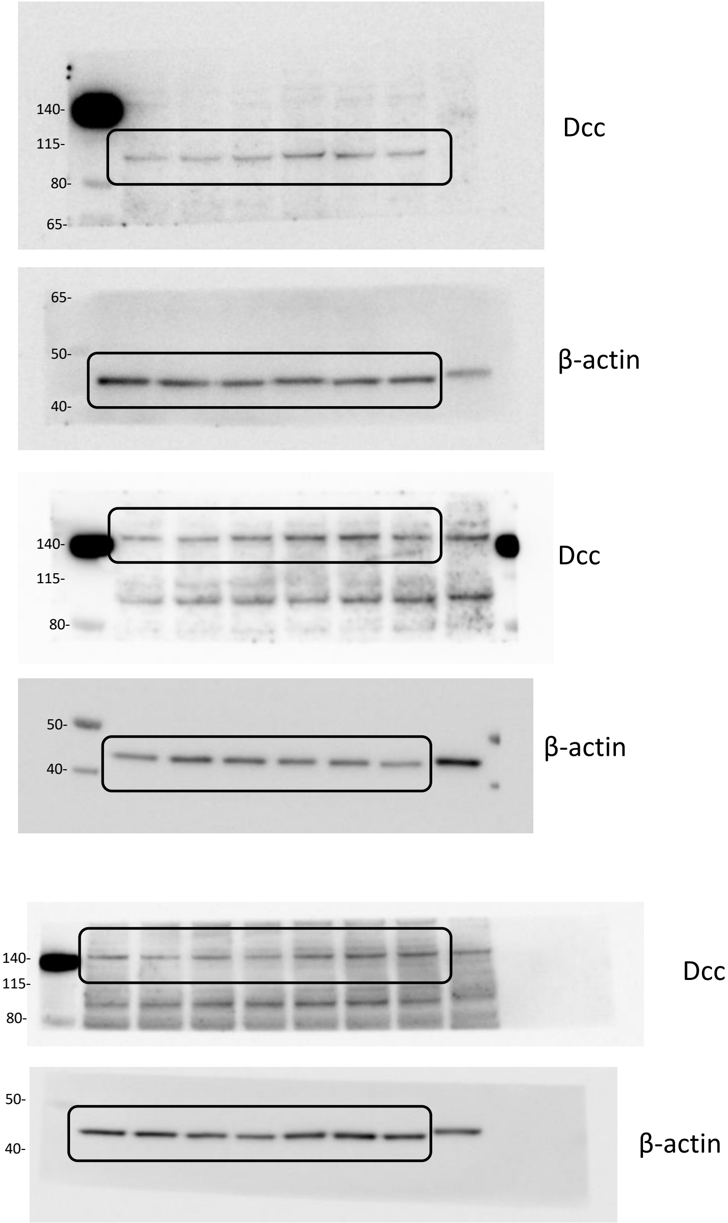
140-
115-
80-
65-
Dcc
65-
50-
40-
β-actin
140-
115-
80-
Dcc
50-
40-
β-actin
140-
115-
80-
Dcc
50-
40-
β-actin

Fig. 3A

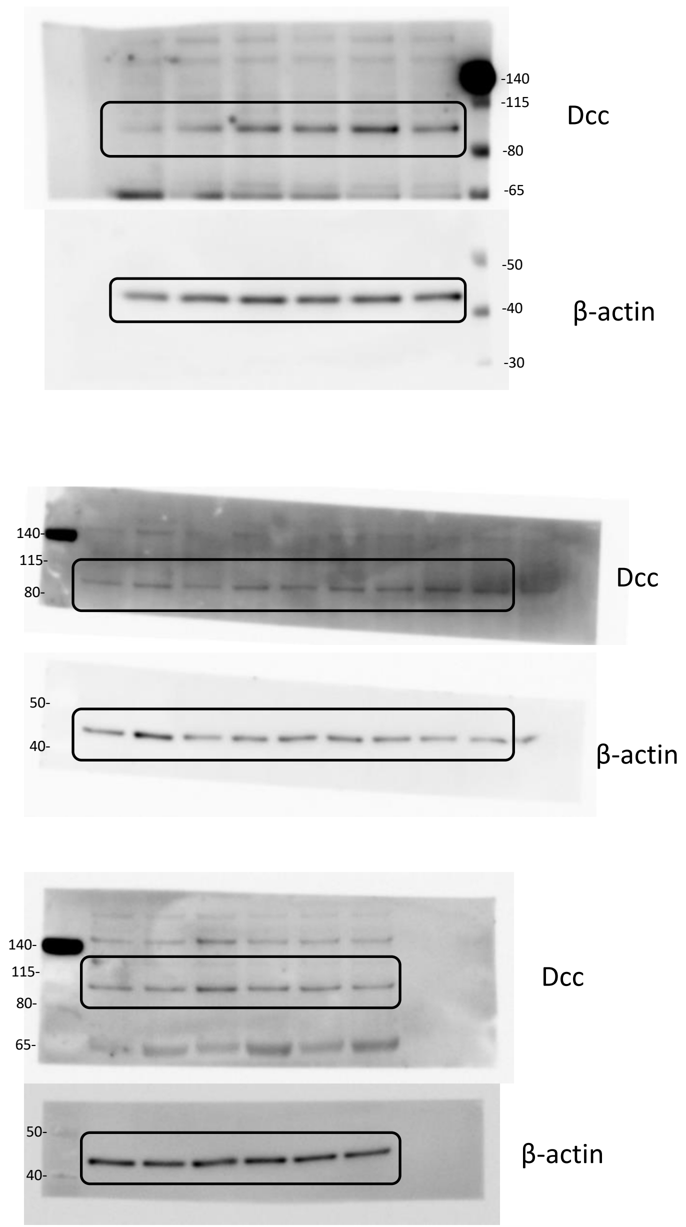
-140
-115
-80
-65
Dcc
-50
-40
-30
β-actin
140-
115-
80-
Dcc
50-
40-
β-actin
140-
115-
80-
65-
Dcc
50-
40-
β-actin

Fig. 4

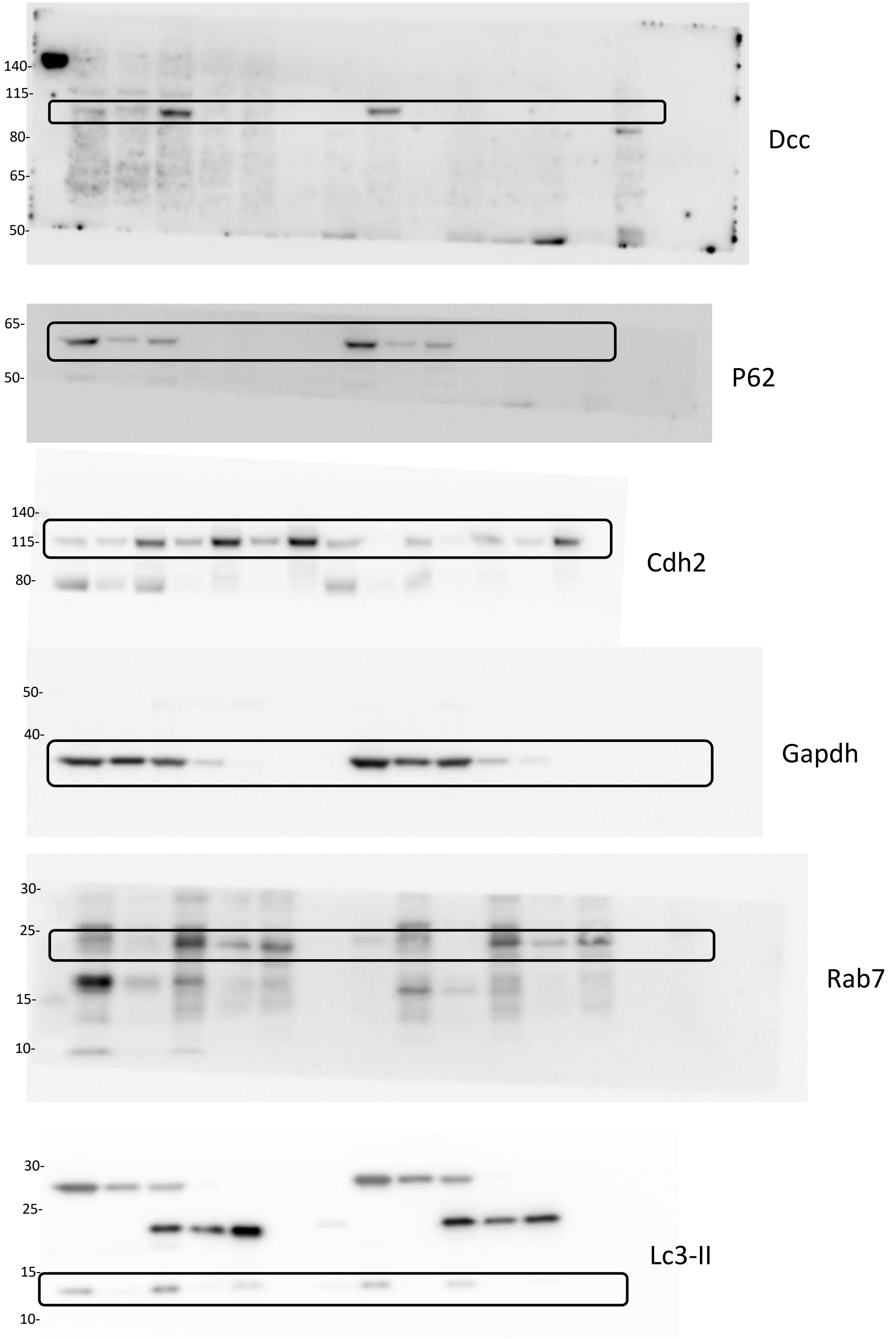

140-
115-
80-
65-
50-
Dcc
65-
50-
P62
140-
115-
80-
Cdh2
50-
40-
Gapdh
30-
25-
15-
10-
Rab7
30-
25-
15-
10-
Lc3-II

Fig. 5A

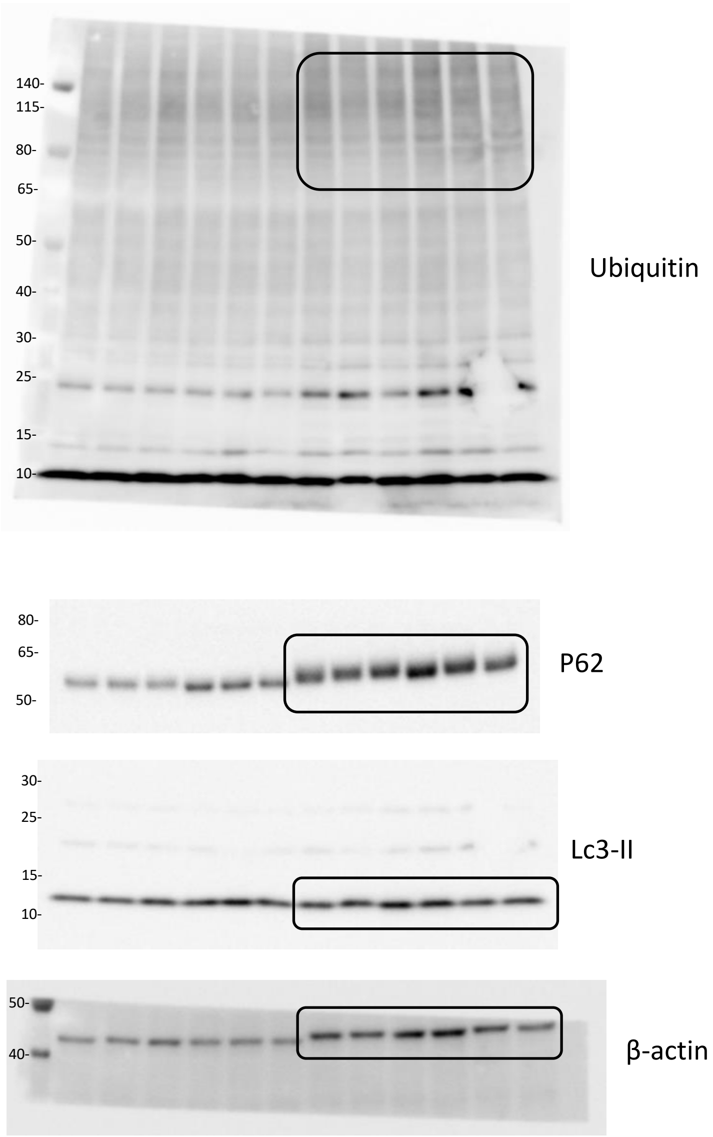
140-
115-
80-
65-
50-
40-
30-
25-
15-
10-
Ubiquitin
80-
65-
50-
P62
30-
25-
15-
10-
Lc3-II
50-
40-
β-actin

Fig. 5B

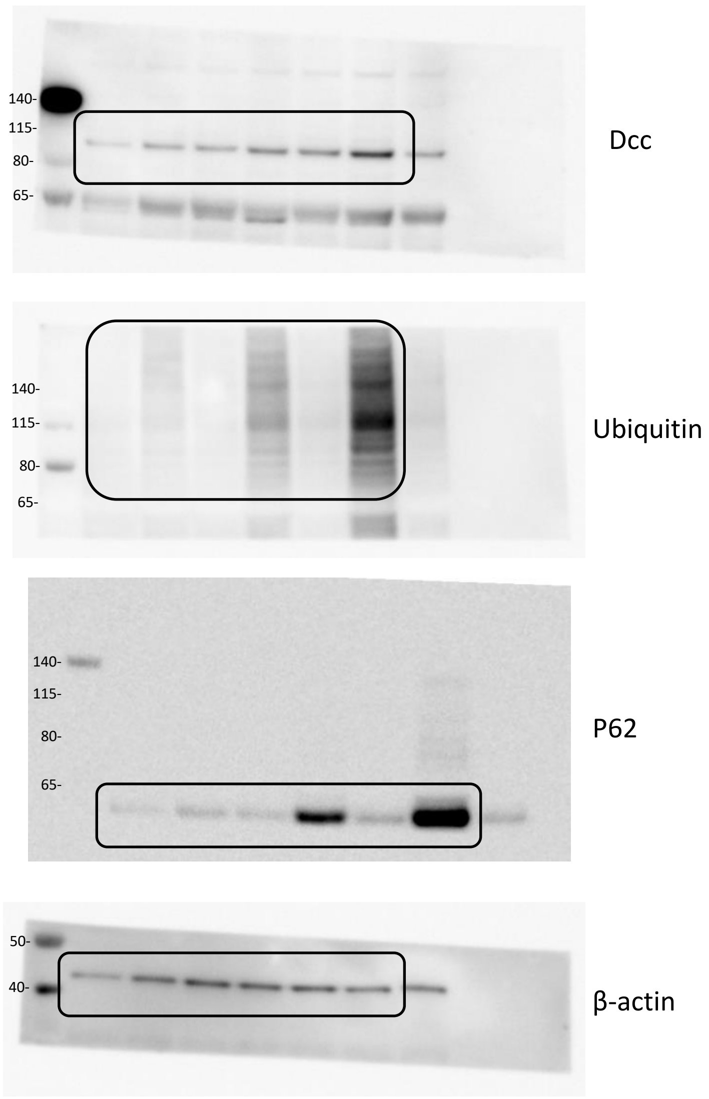

140-
115-
80-
65-
Dcc
140-
115-
80-
65-
Ubiquitin
140-
115-
80-
65-
P62
50-
40-
β-actin

Supplementary Fig. 2A

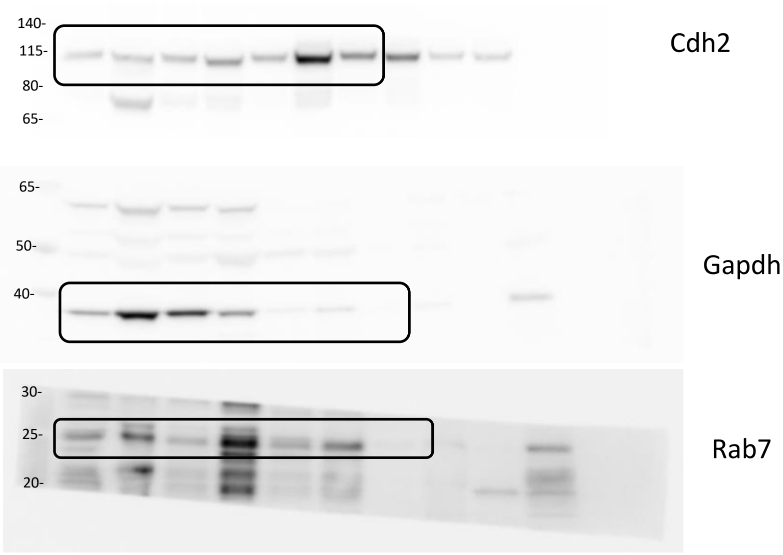

Supplement: Supplementary file 1 — Supplementary material Manzoli et al., 2024 [file 41419_2024_6661_MOESM1_ESM.pdf]
